# Sleep Problems in Childhood and Borderline Personality Disorder Symptoms in Early Adolescence

**DOI:** 10.1007/s10802-016-0158-4

**Published:** 2016-04-25

**Authors:** Suzet Tanya Lereya, Catherine Winsper, Nicole K. Y. Tang, Dieter Wolke

**Affiliations:** 1 Department of Psychology, University of Warwick, Coventry, UK; 2 Division of Mental Health and Wellbeing, Warwick Medical School, University of Warwick, Coventry, UK; 3 Department of Psychology and Division of Mental Health and Wellbeing, University of Warwick, Coventry, UK

**Keywords:** Borderline personality disorder, BPD, Nightmares, Adolescence, Childhood, ALSPAC, Sleep

## Abstract

Sleep disorders, such as insomnia and nightmares, are commonly associated with Borderline Personality Disorder (BPD) in adulthood. Whether nightmares and sleep-onset and maintenance problems predate BPD symptoms earlier in development is unknown. We addressed this gap in the literature using data from the Avon Longitudinal Study of Parents and Children (ALSPAC). Participants included 6050 adolescents (51.4 % female) who completed the UK Childhood Interview for DSM-IV BPD at 11 to 12 years of age. Nightmares and sleep onset and maintenance problems were prospectively assessed via mother report when children were 2.5, 3.5, 4.8 and 6.8 years of age. Psychopathological (i.e., emotional temperament; psychiatric diagnoses; and emotional and behavioural problems) and psychosocial (i.e., abuse, maladaptive parenting, and family adversity) confounders were assessed via mother report. In logistic regressions, persistent nightmares (i.e., regular nightmares at 3 or more time-points) were significantly associated with BPD symptoms following adjustment for sleep onset and maintenance problems and all confounders (Adjusted Odds Ratio = 1.62; 95 % Confidence Interval = 1.12 to 2.32). Persistent sleep onset and maintenance problems were not significantly associated with BPD symptoms. In path analysis controlling for all associations between confounders, persistent nightmares independently predicted BPD symptoms (Probit co-efficient [β] = 0.08, *p* = 0.013). Emotional and behavioural problems significantly mediated the association between nightmares and BPD (β =0.016, *p* < 0.001), while nightmares significantly mediated associations between emotional temperament (β = 0.001, *p* = 0.018), abuse (β = 0.015, *p* = 0.018), maladaptive parenting (β = 0.002, *p* = 0.021) and subsequent BPD. These findings tentatively support that childhood nightmares may potentially increase the risk of BPD symptoms in early adolescence via a number of aetiological pathways. If replicated, the current findings could have important implications for early intervention, and assist clinicians in the identification of children at risk of developing BPD.

**Electronic supplementary material:**

The online version of this article (doi:10.1007/s10802-016-0158-4) contains supplementary material, which is available to authorized users.

Borderline Personality Disorder (BPD) is a serious mental illness characterised by severe behavioural and emotional dysregulation, high rates of comorbid mental disorder and suicidality (Crowell et al. 2009). The aetiology of BPD is only partly known. Recently, researchers have focused on the developmental precursors of BPD in recognition of the fact that personality disorders do not appear *de novo* in adulthood. Rather, they have an identifiable phenotype emergent in childhood or early adolescence (Crowell et al. 2009; Geiger and Crick 2010), which indicates a heightened risk of personality disorder in adulthood (Lahey et al. 2005; Winsper et al. 2016; 2015).

A comprehensive understanding of the pathways and precursors to BPD is essential to elucidate the complex aetiology of this disorder (Cicchetti and Crick 2009), aid the identification of at-risk children, and inform early intervention approaches to prevent the full development of the disorder (Chanen et al. 2007a). Such research is particularly important in view of the reported reluctance of clinicians to diagnose BPD in adolescence (Laurenssen et al. 2013), which means that young people with the disorder risk being misdiagnosed and receiving inappropriate treatment, e.g., pharmacotherapy (Paris 2013).

While there is an expanding body of literature examining developmental pathways to BPD in child and adolescent populations (Belsky et al. 2012; Bornovalova et al. 2013; Cicchetti et al. 2014; Winsper et al. 2014), to our knowledge there are no existing studies examining sleep problems as a potential precursor. This is somewhat surprising considering the centrality of dysregulation to the development and symptom profile of this complex disorder (Crowell et al. 2009; Fleischer et al. 2012; Kaess et al. 2014). Recent reviews report that sleep fragmentation, alterations in Slow Wave Sleep and REM sleep, and dysphoric dreaming are prevalent in adult BPD (Hafizi 2013; Simor and Horváth 2013). Further, adult studies indicate that reduced sleep quality (Plante et al. 2013) and nightmares (Semiz et al. 2008) worsen symptom course. What cannot be ascertained from these cross-sectional studies, however, is whether sleep problems predate the development of BPD or are a consequence of the disorder. In order to determine whether sleep problems may precede the development of BPD, we need studies which prospectively assess sleep problems (in addition to other risk factors) early in childhood (Winsper and Tang 2014).

There are several mechanisms via which sleep problems could be associated with the development of BPD. First, they could represent a mediator (i.e., link in a causal chain) of the relationship between early risk factors and subsequent BPD. Traumatic childhood experiences (e.g., abuse or maladaptive parenting), for example, could increase the risk of nightmares, subsequently increasing the risk of BPD symptoms via alterations to the Hypothalamic Pituitary Axis: HPA (Hellhammer et al. 2009; Rinne et al. 2002). In another mechanism, an overly-emotional temperament could heighten the risk of sleep problems (Owens-Stively et al. 1997; Simard et al. 2008), which may then exacerbate emotional dysregulation increasing the likelihood of BPD (Selby et al. 2013). Second, sleep problems could indirectly increase the risk of BPD by increasing the risk of emotional and behavioural dysregulation (Selby et al. 2013), which if chronic could eventuate in a constellation of BPD symptoms (Crowell et al. 2009).

Over the past 10 to 15 years, a number of studies have demonstrated that sleep problems in childhood are associated with a range of emotional (e.g., depression, anxiety) and behavioural (e.g., attention and conduct, substance use) disorders (see Gregory and Sadeh 2012 for a review). Many, however, have used multicomponent sleep scales conflating sleep problems (i.e., hard to get to sleep, frequent waking, early waking, sleeps more than most children, sleep less than most children, talks or walks in sleep, overtired) with nightmares, making it difficult to ascertain the independent effects of each (Goodnight et al. 2007; Gregory et al. 2005; 2004; Gregory and O’Connor 2002). The few studies specifically considering nightmares demonstrate cross-sectional or longitudinal associations with difficult temperament (Simard et al. 2008); hyperactivity (Li et al. 2011; Schredl et al. 2009); temper outbursts (Li et al. 2011); mood disturbance (Coulombe et al. 2010; Li et al. 2011; Nielsen et al. 2000; Schredl et al. 2009); anxiety (Nielsen et al. 2000); and self-harm (Singareddy et al. 2013).

Similarly, prospective associations between individual sleep problems and psychopathological outcomes have been reported, though assessment tools across studies have lacked consistency (Gregory and Sadeh 2012). Shanahan et al. (2014) found that “difficulty falling asleep” significantly predicted General Anxiety Disorder (GAD)/Depression and Oppositional Defiant Disorder (ODD), while “waking in the middle of the night” significantly predicted ODD. Touchette et al. (2009) reported significant bi-directional associations between short night time sleep duration and hyperactivity trajectories across early childhood. Steinsbekk and Wichstrøm (2015) found that insomnia (DSM-IV criteria) increased risk of developing conduct disorder, major depressive disorder and social phobia, while Attention Deficit Hyperactivity Disorder, Major Depressive Disorder (MDD) and ODD predicted insomnia. As emotional, behavioural and social problems are precursors to (and core features of) youth BPD (Chanen et al. 2007b), collectively these studies suggest that sleep problems may be linked to the development of BPD in young populations.

## The Present Study

In the current study we aimed to examine the prospective associations between nightmares and sleep onset and maintenance problems during childhood and BPD symptoms at 11 to 12 years. Specifically, we explored the following questions: 1) Are sleep (i.e., nightmares, sleep onset, and sleep maintenance) problems independently associated with an increased risk of BPD symptoms following adjustment for important confounders; 2) Do sleep problems mediate the associations between early risk factors (i.e., family adversity, emotional temperament, abuse, and maladaptive parenting) and subsequent BPD; and 3) Do sleep problems indirectly increase the risk of BPD via an increased risk of emotional and behavioural dysregulation? We also examined whether each sleep problem independently predicted BPD (e.g., when testing nightmare-BPD associations we additionally controlled for sleep onset and maintenance problems).

## Method

### Participants

The Avon Longitudinal Study of Parents and Children (ALSPAC) is a UK birth cohort examining the determinants of development, health and disease during childhood and beyond. The study has been described elsewhere (Boyd et al. 2013). ALSPAC recruited pregnant women in Avon with expected dates of delivery between the 1st April 1991 and 31st December 1992. 14,541 pregnant women were initially enrolled in the study, and had returned at least one questionnaire or attended a “Children in Focus” clinic by the 19th July 1999. Of these *initial* pregnancies, there were 14,676 foetuses, resulting in 14,062 live births of which 13,988 children were alive at 1 year of age. When the oldest children were approximately 7 years old, the sample was bolstered with eligible cases who had failed to join the study originally. Consequently, when considering variables collected from the age of seven onwards there are data available on 14,701 children (an additional 713 children). The ALSPAC study website contains details of all of the data that are available through a fully searchable data dictionary (ALSPAC: www.alspac.bris.ac.uk). From the first trimester of pregnancy parents have completed postal questionnaires about the study child’s health and development. The child has attended annual assessment clinics, including face-to face interviews, psychological and physical tests. Ethical approval was obtained from the ALSPAC Law and Ethics committee and the local research ethics committees.

**Fig. 1:**
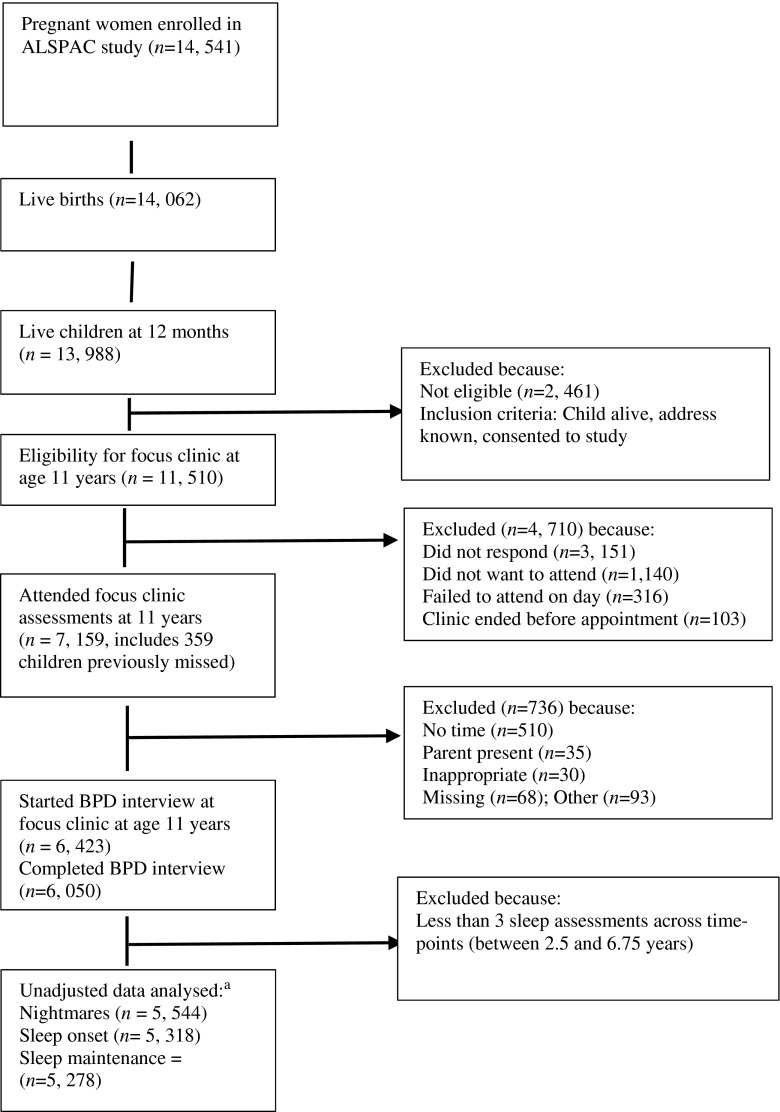
Flow chart of participant numbers from pregnancy to BPD assessment at 11 to 12 years. ^a^ Adjusted numbers reported in Table 3

This study is based on adolescents (mean age 11.8 years) with BPD outcome data (*N* = 6, 050) and sleep assessments (i.e., nightmares, and sleep onset and maintenance problems) across at least three (out of a maximum of four) time-points (Fisher et al. 2013). Numbers included in analyses are presented in Fig. 1 and Table 3 **.**

### Measures

#### BPD Symptoms

Adolescents were interviewed to assess their experience of BPD symptoms over the past 2 years. Trained psychologists assessed BPD using a face-to-face semi-structured interview: the UK Childhood Interview for DSM-IV Borderline Personality Disorder [UK-CI-BPD] (Zanarini et al. 2004). The UK-CI-BPD is based on the borderline module of the Diagnostic Interview for DSM-IV Personality Disorders (Zanarini et al. 1996), which is a widely used semi-structured interview for all DSM-IV Axis II disorders. The inter-rater and test–retest reliability of the DSM-III, DSM-III-R and DSM-IV versions of this measure are good to excellent (Zanarini and Frankenberg 2001). The UK-CI-BPD was adapted from the CI-BPD (US version). The convergent validity of the CI-BPD has been demonstrated: CI-BPD diagnosis was found to be significantly associated with clinician diagnosis, and other measures of BPD (i.e., Borderline Personality Features Scale, Personality Assessment Inventory - Borderline subscale) as reported by patients and parents (Sharp et al. 2012). The inter-rater reliability (k) of the UK-CI-BPD within the current sample was assessed with taped interviews of a sub-group of 30 children. IRR ranged from 0.36 to 1.0 (median value 0.88). 86 % of the k values were within the excellent range of >0.75 (Zanarini et al. 2011).

The UK-CI-BPD comprises nine sections: intense inappropriate anger; affective instability; emptiness; identity disturbance; paranoid ideation; abandonment; suicidal or self-mutilating behaviours; impulsivity and intense unstable relationships. Once a trained assessor had explored each section, a judgment was made as to whether each symptom was definitely present, probably present or absent. A symptom was classed as definitely present if it had occurred very frequently (i.e., daily or at least 25 % of the time), and probably present if it had occurred repeatedly, but did not meet the criterion for definitely present. The derived dichotomous outcome was based on previous studies (Wolke et al. 2012), and represented the very frequent or repeated occurrence of five or more BPD symptoms. Although BPD is sometimes assessed dimensionally in young populations (Crick et al. 2005), we elected to use a dichotomous outcome because we were interested in assessing the risk of sleep problems on the development of BPD symptoms that cross the established clinical threshold (i.e., five or more symptoms). This approach generates results that are easier for interpretation and more relevant to risk screening in clinical settings.

#### Sleep Problems

Nightmares and sleep onset and maintenance problems were assessed by postal questionnaire when the child was 2.5, 3.5, 4.8 and 6.8 years of age. Mothers were asked: “In the past year has your child regularly had nightmares?” (i.e., nightmares); “In the past year, has your child regularly woken in the night?” (i.e., sleep maintenance problems); “In the past year, has your child regularly had difficulty going to sleep?” (i.e., sleep onset problems). We dichotomised each sleep problem response into any sleep problem (i.e., “yes not worried,” “yes bit worried,” and “yes very worried” = 1) versus no sleep problem (i.e., “no” = 0), as we were interested in capturing whether the child had experienced a sleep problem at each time-point, rather than the mothers’ thoughts or concerns regarding their child’s sleep problem. As has been done in previous studies (Fisher et al. 2013) we constructed categorical (e.g., no nightmares; 1 time period; 2 time periods; 3 or more time periods) sleep problem variables to assess the impact of persistent sleep problems.

#### Sex

Sex (male: 48.6 %; female: 51.4 %) was incorporated as a confounder into the analysis.

#### Family Adversity

As family adversity has been previously associated with BPD (Winsper et al. 2012b), it was included in the analysis as a confounder. The Family Adversity Index (FAI) was devised to represent multiple family risk factors, and comprises 18 (15 for the short form) interrelated items covering aspects of early parenthood, housing, financial difficulties, education, partner and family problems, social network, psychopathology of mother, substance abuse and crime. Mothers reported on family adversity via postal questionnaires during pregnancy (18 items), 0 to 2 years (15 items), and 2 to 4 years (15 items). Each validated adversity item was recorded as 1 point and points were then summed to create a total FAI score for each time-point. The total FAI scores for the three time-points were summed and entered into the analysis as a continuous variable in line with recommended use (Bowen et al. 2005) and previous studies (Wolke et al. 2012). The mean family adversity score was 3.76 (SD: 3.87).

#### Emotional Temperament

Emotional temperament has been associated with sleep problems (Owens-Stively et al. 1997) and BPD (Stepp et al. 2014). Mothers completed the Carey Temperament Scale: CTS (Carey and McDevitt 1978) when children were 2 years old. The Mood and Intensity subscales of the CTS were chosen as they map most closely onto emotional temperament as described in the sleep (Simard et al. 2008) and BPD (Crowell et al. 2009; Linehan and Koerner 1993) literature. Mothers were presented with statements describing certain behaviours representing mood (12 items, e.g., “child is fussy on waking up”) and intensity (9 items, e.g., “child shows strong reactions to failure”), and were asked to rate how often their child behaves that way on a scale of 1 (almost never) to 5 (almost always). Total scores from the mood and intensity scales were summed. The mean emotional temperament score was 39.2 (SD: 8.3).

#### Abuse

Abuse experiences are linked with sleep problems (Semiz et al. 2008) and BPD (Widom et al. 2009). Physical and sexual abuse were each assessed with one item (i.e., “he/she was sexually abused” and “he/she was physically abused”) included in the *upsetting events questionnaire* completed by the mother when the child was 2.5, 3.5, 4.8, and 6.8 years old. Consistent with previous research (Winsper et al. 2012a) abuse was classified as present if sexual or physical abuse was reported at any one of the time points.

#### Maladaptive Parenting

Maladaptive parenting is implicated in the development of BPD (Johnson et al. 2000) and childhood sleep problems (Owens-Stively et al. 1997). The maladaptive parenting variable was constructed by using mother-reported indicators of maternal hitting (2, 3.5 and 6.4 years) and hostility (2, 4 & 7 years). Hitting was coded at 2 and 3.5 years on a scale of 0 to 4 (never, rarely, once a week, several times a week, and every day) and at 6.4 years on a scale 0 to 2 (never, sometimes, often) with higher scores representing greater frequency of hitting. Consistent with previously derived scales (Wolke et al. 2012), we created an overall hitting variable by summing the three scales to produce a score from 0 to 10 (mean = 2.96; SD = 1.98). Hostility was indicated by 4 interrelated items, e.g., “I often get very irritated with this child” (Waylen et al. 2008) at 2 and 4 years, and 3 items at 7 years. As in previous research (Wolke et al. 2012), these items were summed to give a total maternal hostility score from 0 to 7 (mean = 1.83; SD = 1.82).

#### DSM-IV Psychiatric Diagnoses

Axis I disorders are associated with adolescent BPD (Chanen et al. 2007b) and sleep problems (Gregory and Sadeh 2012; Jacobs-Rebhun et al. 2000). DSM-IV psychiatric diagnosis according to parent and teacher report were recorded at 7.5 years using the Development and Wellbeing Assessment (DAWBA) (Goodman et al. 2000). The DAWBA has been validated for clinical diagnosis (see http://www.dawba.com/). Any Axis I diagnosis of ADHD, conduct disorder, oppositional defiant disorder, depression or anxiety versus no diagnosis were computed (Fisher et al. 2013).

#### Emotional and Behavioural Problems

Emotional and behavioural problems are strongly associated with sleep disturbance (Schredl et al. 2009) and BPD (Eaton et al. 2011). Mother-reported emotional and behavioural problems were assessed at 9.5 and 11.7 years with the Strengths and Difficulties Questionnaire (Goodman 2001). Variables for each time-point were constructed by summing scores of negative emotionality, hyperactivity, and conduct problems (Lereya et al. 2013). At 9.5 years, Cronbach alphas were 0.68 for the 5 negative emotionality items (e.g., “Child is often unhappy, downhearted or tearful,” “Child is nervous or clingy in new situations”); 0.77 for the 5 hyperactivity items (e.g., “Child is easily distracted,” “Child is constantly fidgeting or squirming); and 0.59 for the 5 conduct problems items (e.g., “Child often lies or cheats,” “Child steals from home, school or elsewhere.”). At 11.7 years, Cronbach alphas were 0.68 for the negative emotionality items, 0.77 for the hyperactivity items, and 0.57 for the conduct problems items.

### Statistical Plan

### Logistic Regression Analysis

The first stage of the analysis was conducted in *SPSS version 21* to ascertain the unadjusted and adjusted associations between persistent sleep problems and subsequent BPD symptoms. We ran logistic regression analyses (using the forced entry method) to assess the associations between: 1) persistent nightmares; 2) sleep onset; and 3) sleep maintenance problems and subsequent BPD symptoms. In Model A, we tested unadjusted associations. In Model B, we included sex; emotional temperament at 2 years; family adversity from pregnancy to 4 years; physical and sexual abuse and maladaptive parenting from 2 to 7 years; DSM-IV diagnosis at 7.5 years; and emotional and behavioural problems at 9.5 and 11.7 years as confounders. In Model C, we additionally controlled for each of the sleep problems not being tested (e.g., when assessing nightmare-BPD associations we additionally controlled for sleep onset and maintenance problems). We also tested the associations between all psychosocial and psychopathological confounders and persistent nightmares in supplementary analysis (we did not conduct supplementary analysis for sleep onset and maintenance problems as they were not significantly associated with BPD symptoms). Results are reported in Odds Ratios (OR) with 95 % Confidence Intervals (CIs).

### Path Analysis

We conducted path analysis using *Mplus version 6* to explicitly examine the complex associations (i.e., direct and indirect) between early risk factors (emotional temperament, sex, family adversity, maladaptive parenting and abuse), persistent nightmares, emotional and behavioural problems at 9.5 years, and subsequent BPD symptoms and emotional and behavioural problems at 11–12 years. As sleep maintenance and onset problems were not significantly associated with BPD and did not attenuate the associations between persistent nightmares and BPD, they were not included in the path analysis.

We used probit estimation as recommended for path analysis with both categorical (e.g., BPD symptoms) and continuous (e.g., emotional and behavioural problems) endogenous variables (Winship and Mare 1983). Probit regression is a log-linear approach analogous to logistic regression producing similar chi-square statistics, *p* values and conclusions to logit models (Allison 1999). Probit regression coefficients indicate the strength of the relationship between the predictor variable and the probability of group membership. They represent the change in the probability of “caseness” associated with a unit change in the independent variable, thus it is important to keep the scale of the predictor in mind when interpreting probit coefficients, i.e., one would expect probit values to be larger for dichotomous predictors, which represent the change from “no caseness” (i.e., no abuse) to “caseness” (i.e., abuse) rather than a single value on a continuous scale (i.e., emotional and behavioural problems scale). The WLSMV estimator (weighted least squares with robust standard errors, mean and variance adjusted) was used yielding probit co-efficients for categorical outcomes and normal linear regression coefficients for continuous outcomes.

### Missing Data

As a substantial proportion of the original sample was lost to follow-up, we conducted a series of logistic regressions to identify significant predictors of attrition. Adolescents lost to attrition were more often boys, of ethnic minority and low birth weight. They more often lived in rented properties, were born to single mothers of lower educational level and had parents with manual jobs (See Table 1). Using the variables associated with selective drop-out as the predictors we fitted a logistic regression model (non-response vs. response as outcome) to determine weights for each individual using the inverse-probability of response (Kinner et al. 2007). We then compared the results from the weighted and unweighted analysis.

**Table 1:** Drop-out analysis comparing those not available for the BPD interview to those who completed the BPD interview

Characteristic	BPD interview not available	BPD interview available	BPD interview not available versus available
	N (%)	N (%)	OR (95 % CI)
Gender			
Male	4282 (54)	2938 (48.6)	[reference]
Female	3644 (46)	3112 (51.4)	**1.25 (1.16, 1.33)**
Ethnicity			
White	5933 (93.8)	5541 (96.2)	[reference]
Black and minority ethnic	393 (6.2)	216 (3.8)	**0.59 (0.50, 0.70)**
Birth weight			
> 2499 g	7344 (93.9)	5707 (95.4)	[reference]
< 2500 g	474 (6.1)	273 (4.6)	**0.74 (0.64, 0.86)**
Marital status			
Single	2186 (30.5)	1095 (18.5)	[reference]
Married	4985 (69.5)	4821 (81.5)	**1.93 (1.78, 2.10)**
Home ownership			
Mortgage	4658 (65)	4901 (83.6)	[reference]
Rent	2510 (35)	958 (16.4)	**0.36 (0.33, 0.40)**
Education of mother			
Below O level	2466 (37.5)	1262 (21.6)	[reference]
O level or above	4113 (62.5)	4577 (78.4)	**2.17 (2.01, 2.36)**
Social class			
Non-manual	2714 (46)	3152 (56.5)	[reference]
Manual	3189 (54)	2430 (43.5)	**0.66 (0.61, 0.71)**
Family adversity (M, SD)	4.35 (4.28)	3.76 (3.87)	**0.96 (0.95, 0.96)**

Boldface indicates significant association at *p* < 0.05

*OR* Odds Ratio, *CI* Confidence Intervals, *M* mean, *SD* standard deviation

## Results

### Sample Characteristics

At age 11 to 12 years, 444 adolescents (7.3 %) had 5 or more BPD symptoms (occurring very frequently or repeatedly). This prevalence is largely comparable to figures reported in a large national representative study of adults, i.e., 5.9 % (Grant et al. 2008) and in a community population of 11 to 14-year-olds: moderate BPD, male: 8.3 %; female: 11.5 %; severe BPD, male: 2.8 %; female: 3.8 % (Bernstein et al. 1993). Frequencies of sleep problems are reported in Table 2. The prevalence of nightmares and sleep-onset problems was high, likely reflecting the age of assessment and sub-clinical threshold for endorsement of sleep problems (Schredl et al. 2009). In contrast the prevalence of sleep maintenance problems was low.

**Table 2:** Frequency of nightmares, and sleep-onset and maintenance problems across time-points

Sleep problem	No time-points	1 time-point	2 time-points	3+ time-points
Nightmares^a^	1403 (25.3 %)	1169 (21.1 %)	943 (17 %)	2029 (36.6 %)
Sleep onset^a^	1159 (21.8 %)	1413 (26.6 %)	1221 (23 %)	1525 (28.7 %)
Sleep maintenance^a^	4135 (78.3 %)	737 (14 %)	297 (5.6 %)	109 (2.1 %)

^a^Defined by mother as “regular” problem

Prospective associations between sleep problems and BPD symptoms are presented in Table 3. The pattern of results from the weighted (using the inverse-probability of response) and unweighted analyses was very similar; therefore, we report the unweighted analysis here.

**Table 3:** Associations between childhood sleep problems and BPD symptoms at 11 to 12 years (unadjusted and adjusted)

	Model A	Model B	Model C
	OR (95 % CI)	OR (95 % CI)	OR (95 % CI)
Nightmares	(*n* = 5544)	(*n* = 4625)	(*n* = 4485)
None	[Reference]	[Reference]	[Reference]
1 time point	1.23 (0.89–1.70)	1.28 (0.86–1.91)	1.27 (0.84–1.93)
2 time points	1.28 (0.91–1.79)	1.21 (0.79–1.84)	1.22 (0.79–1.89)
3 + time points	**1.67 (1.26–2.20)**	**1.56 (1.11–2.20)**	**1.62 (1.12–2.32)**
Sleep maintenance	(*n* = 5278)	(*n* = 4492)	(*n* = 4485)
None	[Reference]	[Reference]	[Reference]
1 time point	1.20 (0.90–1.61)	1.15 (0.82–1.61)	1.09 (0.77–1.54)
2 time points	1.39 (0.92–2.10)	1.25 (0.77–2.03)	1.15 (0.70–1.87)
3 + time points	1.27 (0.63–2.53)	1.19 (0.53–2.66)	1.09 (0.49–2.46)
Sleep onset problems	(*n* = 5318)	(*n* = 4516)	(*n* = 4485)
None	[Reference]	[Reference]	[Reference]
1 time point	1.05 (0.77–1.44)	1.05 (0.72–1.52)	1.01 (0.69–1.48)
2 time points	0.95 (0.68–1.32)	0.79 (0.53–1.19)	0.73 (0.48–1.15)
3 + time points	**1.37 (1.02–1.84)**	1.18 (0.82–1.70)	1.06 (0.72–1.54)

Boldface type indicates significant associations at *p* < 0.05. Model A = Crude Analysis; Model B = Analysis controlling for sex, emotional temperament at 2 years, Family Adversity Index (FAI; pregnancy, 0–2 & 2–4 years), physical or sexual abuse at 2.5, 3.5, 4.8, or 6.8 years, preschool maladaptive parenting, Development and Well-Being Assessment (DAWBA) at 7 years, and emotional and behavioural problems assessed with the Strengths and Difficulties Questionnaire (SDQ) at 9.5 and 11.7 years; Model C = For nightmares: additionally controlling for preschool and school sleep onset problems (assessed at 2.5, 3.5, 4.8, or 6.8 years) and persistent sleep maintenance problems; for sleep onset problems: additionally controlling for nightmares and sleep maintenance problems; for sleep maintenance problems additionally controlling for nightmares and sleep onset problems

*BPD* Borderline personality disorder, *OR* odds ratio, *CI* confidence interval

### Logistic Regression Results

Having persistent nightmares (i.e., regular nightmares during 3 or more time-points) between 2.5 and 6.8 years was significantly associated with BPD symptoms at 11 to 12 years, even after controlling for psychopathological and psychosocial confounders. Persistent sleep onset problems were significantly associated with BPD symptoms, though this association become non-significant following control for confounders. Sleep maintenance problems were not significantly associated with BPD symptoms in unadjusted or adjusted analysis.

Associations for individual time periods (e.g., 2.5 years) are reported in Supplementary Table 1. Associations between persistent nightmares and psychopathological and psychosocial confounders are reported in Supplementary Table 2. Persistent nightmares were significantly associated with emotional temperament in infancy; family adversity from pregnancy to 4 years; DSM-IV axis I psychiatric diagnosis at 7.5 years; emotional and behavioural problems at 9.5 and 11.7 years; and abuse, maladaptive parenting, and sleep-onset and maintenance problems across early childhood.

### Path Analysis

We conducted a path model with BPD symptoms and emotional and behavioural problems at 11 to 12 years as the two main endogenous (i.e., outcome) variables. Based on the extant literature, we incorporated direct associations between all early risk factors (i.e., sex, family adversity, emotional temperament, maladaptive parenting, and abuse), persistent nightmares, emotional and behavioural problems at 9.5 years, and the two outcome variables (BPD symptoms and emotional and behavioural problems at 11 to 12 years). Nightmares were incorporated as an ordinal variable consistent with the observed dose response relationship in the unadjusted analysis. In Mplus, an ordinal variable is treated as a continuous latent variable that exceeds thresholds to give the various outcome categories. One coefficient per ordinal variable is produced. This can be interpreted in the same way as a continuous variable. We modelled direct associations between early risk factors and persistent nightmares and emotional and behavioural problems at 9.5 years. We also modelled indirect (i.e., mediational) associations between early risk factors and the two main outcome variables via persistent nightmares and emotional and behavioural problems at 9.5 years. Indirect associations are automatically computed in Mplus through the multiplication of the two direct- effect coefficients comprising the indirect pathway. For example, abuse significantly predicted nightmares (coefficient = 0.19) and nightmares significantly predicted BPD (co-efficient =0.08); therefore, the indirect association from abuse to BPD via nightmares was 0.19 × 0.08 = 0.015. The standard error of the indirect effect is automatically computed using the Delta method (Bollen and Stine 1990).

**Fig. 2:**
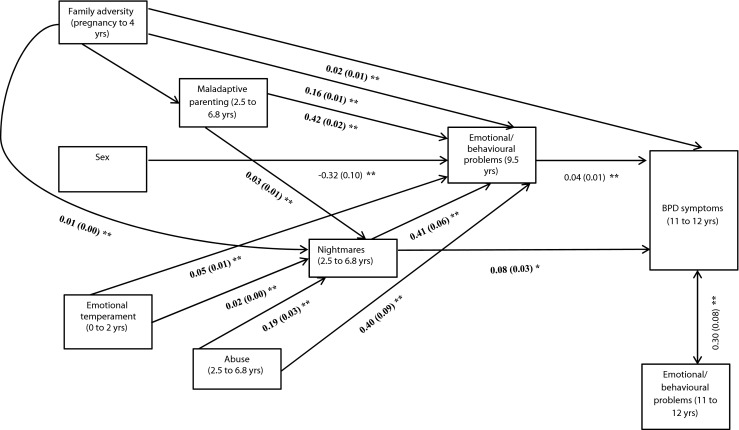
Path diagram showing the main significant direct associations in the final model. Pathways to emotional/behavioural problems at 11 to 12 not shown for clarity; * = significant at *p* < 0.05; ** = significant at *p* < 0.001; Results presented in probit co-efficients; Probit co-efficients represent the change in the probability of “caseness” associated with a unit change in the independent variable, thus it is important to keep the scale of the predictor in mind when interpreting probit coefficients i.e., one would expect probit values to be larger for dichotomous predictors, which represent the change from ‘no caseness’ (i.e., no abuse) to ‘caseness’ (i.e., abuse) rather than a single value on a continuous scale (i.e., emotional/behavioural problems)

The indirect association between persistent nightmares and BPD via emotional and behavioural problems at 9.5 years was also modelled. Thus, sex, family adversity and emotional temperament were incorporated as exogenous (independent) variables, persistent nightmares and emotional and behavioural problems at 9.5 years were included as independent, mediating and endogenous variables, and BPD and emotional and behavioural problems at 11 to 12 years as endogenous variables. DSM-IV axis I psychiatric diagnosis at 7.5 years was excluded from the final path model, as the high degree of overlap between this variable and the emotional and behavioural problems variables appeared to cause multicollinearity issues (i.e., the positive association between DSM-IV diagnosis and BPD outcome reversed becoming negative in the multiple logistic regression). Exclusion of this variable from the path model led to an improved (and acceptable) model fit: χ^2^ = 17.47; *p* < 0.001; RMSEA = 0.038; CFI = 0.99). Figure 2 shows the main significant direct associations in the final model. The main indirect associations are reported in Table 4. Other direct and indirect associations within the model (i.e., to emotional and behavioural problems at 11 to 12 years) are reported in Supplementary Table 3.

**Table 4:** Unstandardised probit coefficients (β) for the indirect pathways between early risk factors, persistent nightmares, and BPD symptoms outcome

Risk factors	To BPD symptoms outcome at 11 to 12 years					
	Associations via Persistent Nightmares at 2.5 to 6.8 yrs			Associations via emotional/behavioural problems at 9.5 years		
	β	SE	*P*	Β	SE	*P*
Sex	0.004	0.003	0.197	**−0.012** ^a^	**0.005**	**0.010**
Emotional temperament	**0.001**	**0.000**	**0.018**	**0.002**	**0.000**	**0.000**
Family adversity	0.001	0.000	0.062	**0.006**	**0.001**	**<0.001**
Abuse	**0.015**	**0.006**	**0.018**	**0.015**	**0.004**	**0.001**
Maladaptive parenting	**0.002**	**0.001**	**0.021**	**0.016**	**0.003**	**<0.001**
Persistent nightmares	N/A	N/A	N/A	**0.016**	**0.004**	**<0.001**

*B* probit coefficient, *SE* standard error, *P* probability

^a^Negative figure indicates male sex as variable coded as 1 = male, 2 = female; Boldface indicates significant associations; Probit co-efficients represent the change in the probability of “caseness” associated with a unit change in the independent variable, thus it is important to keep the scale of the predictor in mind when interpreting probit coefficients i.e., one would expect probit values to be larger for dichotomous predictors, which represent the change from ‘no caseness’ (i.e., no abuse) to ‘caseness’ (i.e., abuse) rather than a single value on a continuous scale (i.e., emotional temperament)

When controlling for all other pathways in the model, persistent nightmares remained a significant predictor of BPD, but not of emotional and behavioural problems at 11 to 12 years. BPD and emotional and behavioural problems at 11 to 12 years were significantly correlated. The association between persistent nightmares and BPD was significantly mediated by emotional and behavioural problems at 9. 5 years. Abuse, maladaptive parenting, emotional temperament and family adversity all remained significant predictors of persistent nightmares. Further, the associations between abuse, maladaptive parenting, emotional temperament and subsequent BPD were all significantly mediated by increased risk of persistent nightmares. In contrast, nightmares did not mediate any of the associations between early risk factors and emotional and behavioural problems at 11 to 12 years.

The indirect associations ranged in magnitude and nature. For example, the indirect effect of persistent nightmares on BPD symptoms via emotional and behavioural symptoms was 0.016, while the direct association was 0.076, thus the indirect effect was approximately a fifth of the direct effect. The indirect effect between maladaptive parenting and BPD via nightmares was weaker representing approximately an eighth of the direct effect. The significant indirect associations between abuse and BPD via nightmares and emotional and behavioural problems at 9.5 years indicated a suppression (i.e., inconsistent mediation) effect (MacKinnon et al. 2000), i.e., nightmares and emotional and behavioural problems at 9.5 years largely explained the association between abuse and BPD.

## Discussion

Using a large birth cohort study, we considered the direct and indirect associations between persistent sleep problems (i.e., nightmares and sleep onset and maintenance problems) in childhood and BPD symptoms at 11 to 12 years. We have three main findings. First, persistent nightmares were independently associated with BPD symptoms at 11 to 12 years. Second, the associations between early risk factors (i.e., emotional temperament, abuse and maladaptive parenting) and BPD were significantly mediated by persistent nightmares. Third, the association between persistent nightmares and BPD was significantly mediated by emotional and behavioural problems at 9.5 years. We will discuss each of these findings in turn.

Persistent nightmares remained a significant predictor of BPD symptoms in multiple logistic regressions controlling for early risk factors (i.e., family adversity, emotional temperament, abuse and maladaptive parenting), DSM-IV psychiatric diagnosis at 7.5 years, preceding (i.e., at 9.5 years) and concurrent (i.e., at 11.7 years) emotional and behavioural problems. In contrast, persistent sleep-onset and maintenance problems were not significantly associated with BPD symptoms. Further, path analysis demonstrated that persistent nightmares significantly predicted BPD but not emotional and behavioural symptoms at 11 to 12 years after controlling for all other associations in the model. Collectively, these findings support that nightmares are associated with an increased risk of BPD symptoms specifically, rather than psychopathology generally. While studies (Coulombe et al. 2010; Schredl et al. 2009; Simard et al. 2008) demonstrate that frequent, chronic nightmares are associated with a range of core BPD features or precursors in youth, to our knowledge this is the first study to demonstrate a prospective link between childhood nightmares and BPD specifically.

In one set of indirect pathways, the associations between early risk factors (i.e., emotional temperament, abuse and maladaptive parenting) and subsequent BPD were significantly mediated by persistent nightmares. While, it should be noted that some of these indirect effects (i.e., involving temperament and maladaptive parenting) were of relatively small magnitude, they are consistent with extant theory, and highlight two potential (and in some cases possibly co-existing) pathways via which nightmares could contribute to the development of BPD. In the first, traumatic events in childhood (i.e., abuse and harsh parenting) could alter Hypothalamic Pituitary Axis (HPA) functioning (Hellhammer et al. 2009) heightening the likelihood of nightmares (van Liempt et al. 2013) and subsequent BPD (Rinne et al. 2002). In the second, an emotional temperament could increase the risk of nightmares, which if persistent, could potentiate dysregulation across time (Selby et al. 2013) until it becomes a trait-like constellation of BPD symptoms (Crowell et al. 2009). Indeed, the final indirect pathway, in which persistent nightmares indirectly increased the risk of BPD via an increased risk of emotional and behavioural dysregulation, may represent a continuation of this process.

Recently, Selby et al. (2013) applied their Emotional Cascades Model (ECM) of BPD to nightmares by demonstrating micro-longitudinal associations between nightmares and subsequent daytime dysregulation in adults with BPD. Viewing this theory through a developmental psychopathology lens (e.g., the bisocial theory, Crowell et al. 2009) may help explain the longitudinal indirect associations observed in the current study. Young individuals with a predisposition towards emotionality (i.e., an emotional temperament) may be more likely to ruminate about upsetting events. In a self-amplifying positive feedback loop, rumination may cause an escalation of negative emotions, leading to further rumination and subsequently, “emotional cascades.” These emotional cascades may spill over into night-time (due to elevated cognitive arousal) increasing risk of nightmares and further dysregulation. Indeed, negatively toned dreams may enhance reactivity to negative emotional stimuli the following day (Lara-Carrasco et al. 2009). Physiologically, nightmares may increase amygdala responsiveness (Nielsen and Stenstrom 2005) and reduce prefrontal cortex activity (Simor et al. 2013) impacting on self-regulatory processes (Dolcos et al. 2006). Cognitively, they may increase the tendency to ruminate (Willner 2004) giving rise to further aversive states during the day (Selby et al. 2013). If this pattern becomes chronic, nightmares could contribute to the exacerbation and maintenance of dysregulation over time, eventually leading to a constellation of precursor BPD traits (Crowell et al. 2009).

It is somewhat surprising that the hypothesised associations between sleep onset and maintenance problems and BPD were not observed. Previous studies suggest associations between sleep disturbances (other than nightmares) and independent BPD features; however, none have assessed associations between BPD psychopathology and sleep onset and maintenance problems specifically. It may be that these problems are not associated with a full constellation of BPD symptoms (i.e., 5 or more) but rather one or two features (Hafizi 2013). The prevalence of sleep maintenance problems was low in the current sample indicating that parents may not have been aware of their child’s frequent night awakenings (Goodnight et al. 2007) unless in the context of nightmares. Due to the novelty of this research area, further prospective studies are required to explicate early aetiological pathways involving well-defined sleep disturbances on the pathway to burgeoning BPD.

Our study has strengths. First, we utilised data from a large prospective cohort with information on a large range of risk factors for both sleep problems and BPD, enabling us to control for a number of relevant confounders and test the potential mechanisms via which nightmares could increase the risk of BPD. Controlling for emotional and behavioural symptoms and co-existing sleep problems enabled us to demonstrate the specificity of the prospective nightmares-BPD association. Second, BPD was assessed using a validated interview. The UK-CI-BPD was adapted from a well validated instrument, was piloted and administered by trained psychologists, and demonstrated high inter-rater reliability (Sharp et al. 2012).

Our study also had limitations. First, there was selective attrition in the cohort, reducing statistical power and potentially biasing results. However, previous simulations indicate that selective drop out may lead to an underestimation of psychiatric disorders but only have a small impact on predictor and outcome relationships (Wolke et al. 2009). Indeed, weighted analysis taking into account factors associated with selective attrition did not substantially alter the results. Nevertheless, drop-out may still limit the generalisability of the results. Second, we could not control for concurrent Post-Traumatic Stress Disorder (PTSD) as this disorder was not assessed at 11 to 12 years. Only one child in the cohort was diagnosed with PTSD at age 7.5 years which may indicate that the nightmare-BPD links observed in this study were not attributable to PTSD. Indeed, Selby et al. (2013) recently reported a significant association between nightmares and BPD independent of PTSD, indicating that nightmares associated with BPD may be qualitatively different to those associated with PTSD (e.g., more related to future concerns such as abandonment issues). Future studies are needed to clarify the independence of the nightmares-BPD link, and may incorporate qualitative assessments to identify themes pertinent to BPD symptoms specifically. Third, as with other epidemiological studies conducted over long time-spans (Gregory et al. 2005), we had to rely on fairly crude caregiver-reported measures of sleep problems and other risk factors, some of which were assessed with just one item. For example, mothers were asked whether their child had “regular” sleep problems, precluding ascertainment of the precise frequency of sleep problems at each time-point. Nevertheless, definitions of persistent sleep problems were based on reports across different time-points, supporting that we were assessing chronic sleep problems. Similarly early childhood risk factors and emotional and behavioural problems were reported by caregivers. This could have led to issues such as an under-reporting of certain behaviours (e.g., maladaptive parenting), though we did find the expected associations between these variables and subsequent nightmares and psychopathology. Future studies may seek to corroborate our findings with other sources of assessment, e.g., multiple-respondents, medical and school records, or direct observation. Fourth, we could not control for the potential bi-directionality of nightmare-BPD associations. Recent studies have indicated that sleep-psychopathology associations may be reciprocal in childhood, though there is more evidence to support that sleep problems precede psychopathology (Shanahan et al. 2014). As we assessed sleep problems from 2.5 years onwards it was not possible to assess pre-existing BPD symptoms. We did, however, control for pre-existing emotional temperament which has been implicated in the development of subsequent nightmares (Simard et al. 2008) and BPD (Stepp et al. 2014).

In summary, we found a robust prospective association between persistent nightmares across early childhood and BPD symptoms at 11 to 12 years. Though we remain tentative in our conclusions, findings suggest that persistent childhood nightmares may be an early indicator and could possibly contribute to the development of BPD via various aetiological pathways. If the current findings are replicated, they could have important implications for clinicians by assisting in the identification of children at risk of developing BPD. More research is now required to clarify the potential mechanisms of intervention for nightmares and BPD during childhood and adolescence, and the extent to which nightmares can be successfully prevented and treated (Fisher et al. 2013). Theoretically, an understanding of how early sleep problems contribute to the aetiology of BPD may be especially salient in view of the centrality of dysregulation to the development of this complex disorder (Crowell et al. 2009).

## Electronic supplementary material

Supplementary Table 1 (DOCX 19 kb)

Supplementary Table 2 (DOCX 18 kb)

Supplementary Table 3 (DOCX 18 kb)

## References

[CR1] Allison PD (1999). Comparing logit and probit coefficients across groups. Sociological Methods & Research.

[CR2] Belsky DW, Caspi A, Arseneault L, Bleidorn W, Fonagy P, Goodman M (2012). Etiological features of borderline personality related characteristics in a birth cohort of 12-year-old children. Development and Psychopathology.

[CR3] Bernstein E, Putnam FW, Ross CA, Torem M, Coons P, Dill D (1993). Validity of the dissociative experiences scale in screening for multiple personality disorder: a multicenter study. American Journal of Psychiatry.

[CR4] Bollen KA, Stine R (1990). Direct and indirect effects: classical and bootstrap estimates of variability. Sociological Methodology.

[CR5] Bornovalova MA, Hicks BM, Iacono WG, McGue M (2013). Longitudinal twin study of borderline personality disorder traits and substance use in adolescence: developmental change, reciprocal effects, and genetic and environmental influences. Personality Disorders: Theory, Research, and Treatment.

[CR6] Bowen E, Heron J, Waylen A, Wolke D (2005). Domestic violence risk during and after pregnancy: findings from a British longitudinal study. BJOG: An International Journal of Obstetrics & Gynaecology.

[CR7] Boyd A, Golding J, Macleod J, Lawlor DA, Fraser A, Henderson J (2013). Cohort profile: the ‘children of the 90s’—the index offspring of the Avon longitudinal study of parents and children. International Journal of Epidemiology.

[CR8] Carey WB, McDevitt SC (1978). Revision of the infant temperament questionnaire. Pediatrics.

[CR9] Chanen AM, McCutcheon LK, Jovev M, Jackson HJ, McGorry PD (2007). Prevention and early intervention for borderline personality disorder. Medical Journal of Australia.

[CR10] Chanen AM, Jovev M, Jackson HJ (2007). Adaptive functioning and psychiatric symptoms in adolescents with borderline personality disorder. Journal of Clinical Psychiatry.

[CR11] Cicchetti D, Crick NR (2009). Precursors and diverse pathways to personality disorder in children and adolescents. Development and Psychopathology.

[CR12] Cicchetti D, Rogosch FA, Hecht KF, Crick NR, Hetzel S (2014). Moderation of maltreatment effects on childhood borderline personality symptoms by gender and oxytocin receptor and FK506 binding protein 5 genes. Development and Psychopathology.

[CR13] Coulombe JA, Reid GJ, Boyle MH, Racine Y (2010). Concurrent associations among sleep problems, indicators of inadequate sleep, psychopathology, and shared risk factors in a population-based sample of healthy Ontario children. Journal of Pediatric Psychology.

[CR14] Crick NR, Murray–Close D, Woods K (2005). Borderline personality features in childhood: a short-term longitudinal study. Development and Psychopathology.

[CR15] Crowell SE, Beauchaine TP, Linehan MM (2009). A biosocial developmental model of borderline personality: elaborating and extending Linehan’s theory. Psychological Bulletin.

[CR16] Dolcos F, Kragel P, Wang L, McCarthy G (2006). Role of the inferior frontal cortex in coping with distracting emotions. Neuroreport.

[CR17] Eaton NR, Krueger RF, Keyes KM, Skodol AE, Markon KE, Grant BF, Hasin DS (2011). Borderline personality disorder co-morbidity: relationship to the internalizing–externalizing structure of common mental disorders. Psychological Medicine.

[CR18] Fisher HL, Lereya ST, Thompson A, Lewis G, Zammit S, Wolke D (2013). Childhood parasomnias and psychotic experiences at age 12 years in a United Kingdom birth cohort. Sleep.

[CR19] Fleischer M, Schäfer M, Coogan A, Häßler F, Thome J (2012). Sleep disturbances and circadian CLOCK genes in borderline personality disorder. Journal of Neural Transmission.

[CR20] Geiger T, Crick N, Ingram RE, Price JM (2010). Developmental pathways to personality disorders. *Vulnerability to psychopathology*, *second edition*: *Risk across the lifespan*.

[CR21] Goodman R (2001). Psychometric properties of the strengths and difficulties questionnaire. Journal of the American Academy of Child & Adolescent Psychiatry.

[CR22] Goodman R, Ford T, Richards H, Gatward R, Meltzer H (2000). The development and well-being assessment: description and initial validation of an integrated assessment of child and adolescent psychopathology. Journal of Child Psychology and Psychiatry.

[CR23] Goodnight JA, Bates JE, Staples AD, Pettit GS, Dodge KA (2007). Temperamental resistance to control increases the association between sleep problems and externalizing behavior development. Journal of Family Psychology.

[CR24] Grant BF, Chou SP, Goldstein RB, Huang B, Stinson FS, Saha TD (2008). Prevalence, correlates, disability, and comorbidity of DSM-IV borderline personality disorder: results from the wave 2 national epidemiologic survey on alcohol and related conditions. The Journal of Clinical Psychiatry.

[CR25] Gregory AM, O’Connor TG (2002). Sleep problems in childhood: a longitudinal study of developmental change and association with behavioral problems. Journal of the American Academy of Child & Adolescent Psychiatry.

[CR26] Gregory AM, Sadeh A (2012). Sleep, emotional and behavioral difficulties in children and adolescents. Sleep Medicine Reviews.

[CR27] Gregory AM, Eley TC, O’Connor TG, Plomin R (2004). Etiologies of associations between childhood sleep and behavioral problems in a large twin sample. Journal of the American Academy of Child & Adolescent Psychiatry.

[CR28] Gregory AM, Caspi A, Eley TC, Moffitt TE, O’Connor TG, Poulton R (2005). Prospective longitudinal associations between persistent sleep problems in childhood and anxiety and depression disorders in adulthood. Journal of Abnormal Child Psychology.

[CR29] Hafizi S (2013). Sleep and borderline personality disorder: a review. Asian Journal of Psychiatry.

[CR30] Hellhammer DH, Wüst S, Kudielka BM (2009). Salivary cortisol as a biomarker in stress research. Psychoneuroendocrinology.

[CR31] Jacobs-Rebhun, S., Schnurr, P. P., Friedman, M. J., Peck, R., Brophy, M., & Fuller, D. (2000). Posttraumatic stress disorder and sleep difficulty. *The American Journal of Psychiatry*, 1525–1526. 10.1176/appi.ajp.157.9.1525 10964881

[CR32] Johnson JG, Smailes EM, Cohen P, Brown J, Bernstein DP (2000). Associations between four types of childhood neglect and personality disorder symptoms during adolescence and early adulthood: findings of a community-based longitudinal study. Journal of Personality Disorders.

[CR33] Kaess M, Brunner R, Chanen A (2014). Borderline personality disorder in adolescence. Pediatrics.

[CR34] Kinner SA, Alati R, Najman JM, Williams GM (2007). Do paternal arrest and imprisonment lead to child behaviour problems and substance use? A longitudinal analysis. Journal of Child Psychology and Psychiatry.

[CR35] Lahey BB, Loeber R, Burke JD, Applegate B (2005). Predicting future antisocial personality disorder in males from a clinical assessment in childhood. Journal of Consulting and Clinical Psychology.

[CR36] Lara-Carrasco J, Nielsen TA, Solomonova E, Levrier K, Popova A (2009). Overnight emotional adaptation to negative stimuli is altered by REM sleep deprivation and is correlated with intervening dream emotions. Journal of Sleep Research.

[CR37] Laurenssen EMP, Hutsebaut J, Feenstra DJ, Van Busschbach JJ, Luyten P (2013). Diagnosis of personality disorders in adolescents: a study among psychologists. Child and Adolescent Psychiatry and Mental Health.

[CR38] Lereya ST, Winsper C, Heron J, Lewis G, Gunnell D, Fisher HL, Wolke D (2013). Being bullied during childhood and the prospective pathways to self-harm in late adolescence. Journal of the American Academy of Child & Adolescent Psychiatry.

[CR39] Li SX, Yu MWM, Lam SP, Zhang J, Li AM, Lai KYC, Wing YK (2011). Frequent nightmares in children: familial aggregation and associations with parent-reported behavioral and mood problems. Sleep.

[CR40] Linehan MM, Koerner K, Paris J (1993). A behavioral theory of borderline personality disorder. *Borderline personality disorder*: *Etiology and treatment*.

[CR41] MacKinnon DP, Krull JL, Lockwood CM (2000). Equivalence of the mediation, confounding and suppression effect. Prevention Science.

[CR42] Nielsen TA, Stenstrom P (2005). What are the memory sources of dreaming?. Nature.

[CR43] Nielsen TA, Laberge L, Paquet J, Tremblay RE, Vitaro F, Montplaisir J (2000). Development of disturbing dreams during adolescence and their relationship to anxiety symptoms. Sleep.

[CR44] Owens-Stively J, Frank N, Smith A, Hagino O, Spirito A, Arrigan M, Alario AJ (1997). Child temperament, parenting discipline style, and daytime behavior in childhood sleep disorders. Journal of Developmental & Behavioral Pediatrics.

[CR45] Paris J (2013). Personality disorders begin in adolescence. Journal of the Canadian Academy of Child and Adolescent Psychiatry.

[CR46] Plante DT, Frankenburg FR, Fitzmaurice GM, Zanarini MC (2013). Relationship between sleep disturbance and recovery in patients with borderline personality disorder. Journal of Psychosomatic Research.

[CR47] Rinne T, De Kloet ER, Wouters L, Goekoop JG, DeRijk RH, van den Brink W (2002). Hyperresponsiveness of hypothalamic-pituitary-adrenal axis to combined dexamethasone/corticotropin-releasing hormone challenge in female borderline personality disorder subjects with a history of sustained childhood abuse. Biological Psychiatry.

[CR48] Schredl M, Fricke-Oerkermann L, Mitschke A, Wiater A, Lehmkuhl G (2009). Longitudinal study of nightmares in children: stability and effect of emotional symptoms. Child Psychiatry and Human Development.

[CR49] Selby EA, Ribeiro JD, Joiner TE (2013). What dreams may come: emotional cascades and nightmares in borderline personality disorder. Dreaming.

[CR50] Semiz UB, Basoglu C, Ebrinc S, Cetin M (2008). Nightmare disorder, dream anxiety, and subjective sleep quality in patients with borderline personality disorder. Psychiatry and Clinical Neurosciences.

[CR51] Shanahan L, Copeland WE, Angold A, Bondy CL, Costello EJ (2014). Sleep problems predict and are predicted by generalized anxiety/depression and oppositional defiant disorder. Journal of the American Academy of Child & Adolescent Psychiatry.

[CR52] Sharp C, Ha C, Michonski J, Venta A, Carbone C (2012). Borderline personality disorder in adolescents: evidence in support of the childhood interview for DSM-IV borderline personality disorder in a sample of adolescent inpatients. Comprehensive Psychiatry.

[CR53] Simard V, Nielsen TA, Tremblay RE, Boivin M, Montplaisir JY (2008). Longitudinal study of bad dreams in preschool-aged children: prevalence, demographic correlates, risk and protective factors. Sleep.

[CR54] Simor P, Horváth K (2013). Altered sleep in borderline personality disorder in relation to the core dimensions of psychopathology. Scandinavian Journal of Psychology.

[CR55] Simor P, Bódizs R, Horváth K, Ferri R (2013). Disturbed dreaming and the instability of sleep: altered nonrapid eye movement sleep microstructure in individuals with frequent nightmares as revealed by the cyclic alternating pattern. Sleep.

[CR56] Singareddy R, Krishnamurthy VB, Vgontzas AN, Fernandez-Mendoza J, Calhoun SL, Shaffer ML, Bixler EO (2013). Subjective and objective sleep and self-harm behaviors in young children: a general population study. Psychiatry Research.

[CR57] Steinsbekk S, Wichstrøm L (2015). Stability of sleep disorders from preschool to first grade and their bidirectional relationship with psychiatric symptoms. Journal of Developmental & Behavioral Pediatrics.

[CR58] Stepp SD, Keenan K, Hipwell AE, Krueger RF (2014). The impact of childhood temperament on the development of borderline personality disorder symptoms over the course of adolescence. Borderline Personality Disorder and Emotion Dysregulation.

[CR59] Touchette E, Côté SM, Petit D, Liu X, Boivin M, Falissard B (2009). Short nighttime sleep-duration and hyperactivity trajectories in early childhood. Pediatrics.

[CR60] van Liempt S, Arends J, Cluitmans PJ, Westenberg HG, Kahn RS, Vermetten E (2013). Sympathetic activity and hypothalamo-pituitary–adrenal axis activity during sleep in post-traumatic stress disorder: a study assessing polysomnography with simultaneous blood sampling. Psychoneuroendocrinology.

[CR61] Waylen A, Stallard N, Stewart-Brown S (2008). Parenting and health in mid-childhood: a longitudinal study. The European Journal of Public Health.

[CR62] Widom CS, Czaja SJ, Paris J (2009). A prospective investigation of borderline personality disorder in abused and neglected children followed up into adulthood. Journal of Personality Disorders.

[CR63] Willner P (2004). Brief cognitive therapy of nightmares and post-traumatic ruminations in a man with a learning disability. British Journal of Clinical Psychology.

[CR64] Winship C, Mare RD (1983). Structural equations and path analysis for discrete data. American Journal of Sociology.

[CR65] Winsper C, Tang NK (2014). Linkages between insomnia and suicidality: prospective associations, high-risk subgroups and possible psychological mechanisms. International Review of Psychiatry.

[CR66] Winsper, C., Lereya, T., Zanarini, M., & Wolke, D. (2012a). Involvement in bullying and suicide-related behavior at 11 years: A prospective birth cohort study. *Journal of the American Academy of Child* & *Adolescent Psychiatry*, *51*, 271–282. e273. 10.1016/j.jaac.2012.01.001 22365463

[CR67] Winsper C, Zanarini M, Wolke D (2012). Prospective study of family adversity and maladaptive parenting in childhood and borderline personality disorder symptoms in a non-clinical population at 11 years. Psychological Medicine.

[CR68] Winsper C, Wolke D, Lereya T (2014). Prospective associations between prenatal adversities and borderline personality disorder at 11–12 years. Psychological Medicine.

[CR69] Winsper C, Marwaha S, Lereya S, Thompson A, Eyden J, Singh S (2015). Clinical and psychosocial outcomes of borderline personality disorder in childhood and adolescence: a systematic review. Psychological Medicine.

[CR70] Winsper C, Lereya ST, Marwaha S, Thompson A, Eyden J, Singh SP (2016). The aetiological and psychopathological validity of borderline personality disorder in youth: a systematic review and meta-analysis. Clinical Psychology Review.

[CR71] Wolke D, Waylen A, Samara M, Steer C, Goodman R, Ford T, Lamberts K (2009). Selective drop-out in longitudinal studies and non-biased prediction of behaviour disorders. The British Journal of Psychiatry.

[CR72] Wolke D, Schreier A, Zanarini MC, Winsper C (2012). Bullied by peers in childhood and borderline personality symptoms at 11 years of age: a prospective study. Journal of Child Psychology and Psychiatry.

[CR73] Zanarini M, Frankenberg F (2001). Attainment and maintenance of reliability of Axis I and II disorders over the course of a longitudinal study. Comprehensive Psychiatry.

[CR74] Zanarini M, Frankenburg F, Sickel A, Yong L (1996). *The Diagnostic Interview for DSM-IV Personality Disorders* (*DIPD-IV*).

[CR75] Zanarini M, Horwood J, Waylen A, Wolke D (2004). The UK version of the childhood interview for DSM-IV borderline personality disorder (UK-CI-BPD).

[CR76] Zanarini M, Horwood J, Wolke D, Waylen A, Fitzmaurice G, Grant B (2011). Prevalence of DSM-IV borderline personality disorder in two community samples: 6,330 English 11-year olds and 34,653 American adults. Journal of Personality Disorders.

