# Supplementary material for: Sleep Problems in Childhood and Borderline Personality Disorder Symptoms in Early Adolescence
Source: J Abnorm Child Psychol. 2016 Apr 25;45(1):193–206. doi: 10.1007/s10802-016-0158-4 (PMC5219009; doi:10.1007/s10802-016-0158-4)
Supplement: Supplementary file 1 — (DOCX 19 kb) [file 10802_2016_158_MOESM1_ESM.docx]

| **Supplementary Table 1:** Childhood Sleep Problems (by year) and Probable/Definite BPD Symptoms at 11 to 12 Years | | | |
| --- | --- | --- | --- |
|  | | **Model A** | **Model B** |
|  |  | **OR (95% CI)** | **OR (95% CI)** |
| **Nightmares – 2.5 years** | | (n = 5398) | (n = 4463) |
| No | | [Reference] | [Reference] |
| Yes | | **1.36 (1.09 – 1.70)** | 1.27 (.99 – 1.64) |
| **Nightmares – 3.5 years** | | (n = 5460) | (n = 4529) |
| No | | [Reference] | [Reference] |
| Yes | | **1.48 (1.20 – 1.82)** | 1.23 (.97 – 1.56) |
| **Nightmares - 4.8 years** | | (n = 5262) | (n = 4425) |
| No | | [Reference] | [Reference] |
| Yes | | **1.27 (1.03 – 1.57)** | **1.28 (1.00 – 1.62)** |
| **Nightmares – 6.8 years** | | (n = 5124) | (n = 4416) |
| No | | [Reference] | [Reference] |
| Yes | | **1.29 (1.05 – 1.60)** | **1.29 (1.01 – 1.64)** |
| **Sleep maintenance problems 2.5 years** | | (n = 5401) | (n = 4466) |
| No | | [Reference] | [Reference] |
| Yes | | 1.18 (.90 – 1.55) | 1.11 (.82 – 1.51) |
| **Sleep maintenance problems 3.5 years** | | (n = 5394) | (n = 4477) |
| No | | [Reference] | [Reference] |
| Yes | | 1.29 (.95 – 1.77) | 1.15 (.80 – 1.67) |
| **Sleep maintenance problems 4.8 years** | | (n = 5269) | (n = 4427) |
| No | | [Reference] | [Reference] |
| Yes | | **1.50 (1.00 – 2.26)** | 1.25 (.78 – 2.00) |
| **Sleep maintenance problems 6.8 years** | | (n = 5059) | (n = 4356) |
| No | | [Reference] | [Reference] |
| Yes | | 1.62 (.83 – 3.15) | 1.08 (.48 – 2.41) |
| **Sleep onset problems - 2.5 years** | | (n = 5385) | (n = 4452) |
| No | | [Reference] | [Reference] |
| Yes | | **1.34 (1.09 – 1.65)** | 1.21 (.95 – 1.54) |
| **Sleep onset problems - 3.5 years** | (n = 5460) | | (n = 4529) |
| No | [Reference] | | [Reference] |
| Yes | **1.30 (1.05 – 1.60)** | | 1.11 (.87 – 1.42) |
| **Sleep onset problems - 4.8 years** | (n = 5310) | | (n = 4459) |
| No | [Reference] | | [Reference] |
| Yes | 1.17 (.95 – 1.44) | | 1.03 (.81 – 1.31) |
| **Sleep onset problems - 6.8 years** | (n = 5155) | | (n = 4438) |
| No | [Reference] | | [Reference] |
| Yes | .97 (.78 – 1.20) | | .89 (.70 – 1.14) |
| Boldface type indicates significant associations at P < 0.05. Model A = Crude Analysis; Model B = Analysis controlling for sex, emotional temperament at 2 years, Family Adversity Index (FAI; pregnancy, 0-2 & 2-4 years), physical or sexual abuse at 2.5, 3.5, 4.8, or 6.8 years, preschool maladaptive parenting, Development and Well-Being Assessment (DAWBA) at 7.5 years, and emotional and behavioural problems assessed with the Strengths and Difficulties Questionnaire (SDQ) at 9.5 years. Abbreviations: BPD, Borderline personality disorder; OR, odds ratio; CI, confidence interval. | | | |
