# Supplementary material for: Sleep Problems in Childhood and Borderline Personality Disorder Symptoms in Early Adolescence
Source: J Abnorm Child Psychol. 2016 Apr 25;45(1):193–206. doi: 10.1007/s10802-016-0158-4 (PMC5219009; doi:10.1007/s10802-016-0158-4)
Supplement: Supplementary file 2 — (DOCX 18 kb) [file 10802_2016_158_MOESM2_ESM.docx]

| **Supplementary Table 2:** Associations between persistent nightmares and psychopathological and psychosocial confounders | | | | |
| --- | --- | --- | --- | --- |
|  | **Persistent Nightmares between Ages 2.5 and 9.5** | | | |
|  | **None** | **1 time point** | **2 time points** | **3 or more time points** |
| **Sex ^1^**  (n = 5544) | [Reference] | .94 (.80 – 1.10) | .97 (.82 – 1.15) | 1.00 (.88 – 1.15) |
| **Emotional Temperament ^2^**  (n = 5269) | [Reference] | **1.03 ( 1.02 – 1.04)** | **1.03 (1.02 – 1.04)** | **1.05 (1.04 – 1.06)** |
| **Family Adversity ^3^**  (n = 5543) | [Reference] | **1.03 (1.00 – 1.05)** | **1.03 (1.01 – 1.05)** | **1.07 (1.05 – 1.09)** |
| **Physical or Sexual Abuse ^4^**  (n = 5544) | [Reference] | **1.66 (1.22 – 2.27)** | **2.49 (1.84 – 3.38)** | **2.84 (2.18 – 3.71)** |
| **Preschool Maladaptive Parenting**  (n = 5478) | [Reference] | **1.04 ( 1.01 – 1.06)** | **1.08 (1.05 – 1.11)** | **1.11 (1.08 – 1.13)** |
| **DSM-IV Diagnosis ^5^**  (n = 5255) | [Reference] | 1.05 (.65 – 1.68) | **1.61 (1.03 – 2.54)** | **2.26 (1.56 – 3.28)** |
| **Emotional and Behavioural**  **Problems at 9.5 yrs ^6^**  (n = 4980) | [Reference] | **1.05 (1.02 – 1.07)** | **1.07 (1.04 – 1.10)** | **1.13 (1.11 – 1.16)** |
| **Emotional and Behavioural**  **Problems at 11.7 yrs ^6^**  (n=4846) | [Reference] | **1.03 (1.00 – 1.06)** | **1.07 (1.04 – 1.10)** | **1.12 (1.10. 1.14)** |
| **Preschool and School Sleep Onset Problems ^7^**  (n = 5316) | [Reference] | **1.28 (1.19 – 1.38)** | **1.50 (1.39 – 1.62)** | **1.99 (1.86 – 2.13)** |
| **Preschool and School Sleep maintenance ^8^**  (n = 5274) | [Reference] | **1.07 (.92 – 1.24)** | **1.37 (1.18 – 1.58)** | **1.87 (1.66 – 2.11)** |
| ^1^ Reference category is being a female; ^2^ emotional temperament measured at 2 years; ^3^ Family Adversity Index (FAI; pregnancy, 0-2 & 2-4 years), ^4^ physical or sexual abuse at 2.5, 3.5, 4.8, or 6.8 years, reference category is not being abused at any time point; ^5^ DSM-IV Diagnosis measured with the Development and Well-Being Assessment (DAWBA) at 7.5 years, ^6^ emotional and behavioural problems assessed with the Strengths and Difficulties Questionnaire (SDQ) at 9.5 and 11.7 years; preschool and school persistent sleep onset problems (assessed at 2.5, 3.5, 4.8, or 6.8 years); and  ^8^ preschool and school persistent sleep maintenance problems (assessed at 2.5, 3.5, 4.8, or 6.8 years) | | | | |
