# Supplementary material for: Sleep Problems in Childhood and Borderline Personality Disorder Symptoms in Early Adolescence
Source: J Abnorm Child Psychol. 2016 Apr 25;45(1):193–206. doi: 10.1007/s10802-016-0158-4 (PMC5219009; doi:10.1007/s10802-016-0158-4)
Supplement: Supplementary file 3 — (DOCX 18 kb) [file 10802_2016_158_MOESM3_ESM.docx]

| **Supplementary Table 3. Unstandardised probit coefficients (β) for the direct and indirect pathways between early risk factors, persistent nightmares, and emotional and behavioural problems outcome** | | | | | | | | | |
| --- | --- | --- | --- | --- | --- | --- | --- | --- | --- |
|  | **Direct pathways to emotional and behavioural problems at 11-12 years** | | | **Indirect pathways to emotional and behavioural problems at 11-12 years** | | | | | |
| **Risk factors** |  |  |  | **Associations via Persistent Nightmares at 2.5 to 6.8 yrs** | | | **Associations via emotional/behavioural problems at 9.5 years** | | |
|  | **β** | **SE** | ***P*** | **β** | **SE** | ***P*** | **β** | **SE** | ***P*** |
| Sex | -0.082^a^ | 0.085 | 0.333 | 0.004 | 0.003 | 0.251 | **-0.192** | **0.063** | **0.002** |
| Temperament | **0.014** | **0.005** | **0.009** | 0.001 | 0.001 | 0.091 | **0.029** | **0.004** | **<0.000** |
| Family adversity | **0.062** | **0.010** | **<0.000** | 0.001 | 0.001 | 0.146 | **0.099** | **0.008** | **<0.000** |
| Abuse | 0.061 | 0.069 | 0.381 | 0.016 | 0.009 | 0.089 | **0.242** | **0.052** | **<0.000** |
| Maladaptive parenting | **0.105** | **0.013** | **<0.000** | 0.003 | 0.002 | 0.097 | **0.254** | **0.010** | **<0.000** |
| Persistent nightmares | 0.081 | 0.047 | 0.084 | N/A | N/A | N/A | **0.252** | **0.035** | **<0.000** |
| Emotional/behavioural problems at 9.5 | 0.611 | 0.010 | **<0.000** | N/A | N/A | N/A | N/A | N/A | N/A |
| B=probit coefficient; SE=standard error; P=probability; ^a^ Negative figure indicates male sex as variable coded as 1=male, 2 = female; Boldface indicates significant associations | | | | | | | | | |
